# Di-μ-oxido-bis­{[(*R*,*R*)-(+)-1-amino-2-(3-methoxy-2-oxidobenzyl­ideneamino-κ^2^ *O* ^2^,*N*)-1,2-diphenyl­ethane-κ*N*]oxidovanadium(V)} dihydrate

**DOI:** 10.1107/S1600536808014396

**Published:** 2008-06-07

**Authors:** Grzegorz Romanowski, Tadeusz Lis

**Affiliations:** a University of Gdańsk, Faculty of Chemistry, Sobieskiego 18/19, 80-952 Gdańsk, Poland; b University of Wroclaw, Faculty of Chemistry, F. Joliot-Curie 14, 50-283 Wroclaw, Poland

## Abstract

In the crystal structure of the title compound, [V_2_(C_22_H_21_N_2_O_2_)_2_O_4_]·2H_2_O, oxide-bridged dimers of the complex are linked to water mol­ecules by hydrogen-bonding inter­actions. The two five-membered chelate rings in the dimeric mol­ecule both adopt twist conformations. Each V^V^ atom is six-coordinated by one oxide group and by two N and one O atom of the tridentate Schiff base ligand, and is bridged by two additional oxide atoms. The metal centre has a distorted octa­hedral coordination. The monoanionic ligands occupy one equatorial and two axial positions.

## Related literature

For general background, see: Robinson *et al.* (1986 ▶); Vilter (1984 ▶); Gruning & Rehder (2000 ▶); Casny & Rehder (2001 ▶); Kimblin *et al.* (2002 ▶); Kwiatkowski *et al.* (2003 ▶, 2007 ▶); Romanowski *et al.* (2008 ▶); Wever & Hemrika (1997 ▶); Butler & Carter-Franklin (2004 ▶). For related structures, see: Root *et al.* (1993 ▶); Romanowski *et al.* (2008 ▶); Colpas *et al.* (1994 ▶); Li *et al.* (1988 ▶). For the synthesis, see: Kwiatkowski *et al.* (2003 ▶).
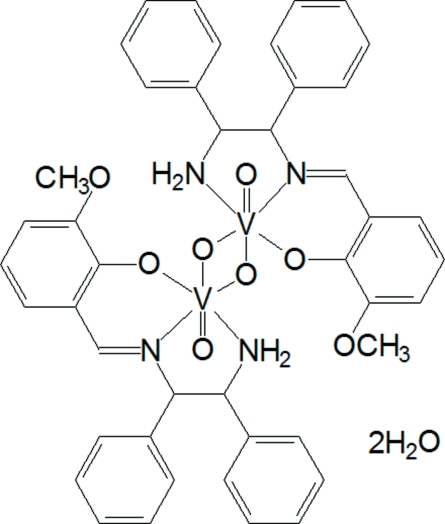


## Experimental

### 

#### Crystal data

[V_2_(C_22_H_21_N_2_O_2_)_2_O_4_]·2H_2_O
*M* *_r_* = 892.73
Orthorhombic, 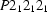

*a* = 9.328 (4) Å
*b* = 16.950 (7) Å
*c* = 25.490 (10) Å
*V* = 4030 (3) Å^3^
*Z* = 4
Cu *K*α radiation
μ = 4.44 mm^−1^
*T* = 100 (2) K
0.14 × 0.12 × 0.09 mm

#### Data collection

Oxford Xcalibur PX diffractometer with CCD area-detector
Absorption correction: analytical (*CrysAlis RED*; Oxford Diffraction, 2006 ▶) *T*_min_ = 0.627, *T*_max_ = 0.768
27320 measured reflections
7083 independent reflections
3677 reflections with *I* > 2σ(*I*)
*R*_int_ = 0.066

#### Refinement

*R*[*F* ^2^ > 2σ(*F* ^2^)] = 0.048
*wR*(*F* ^2^) = 0.069
*S* = 1.01
7083 reflections
541 parameters
H-atom parameters constrained
Δρ_max_ = 0.64 e Å^−3^
Δρ_min_ = −0.39 e Å^−3^
Absolute structure: Flack (1983 ▶), 2905 Friedel pairs
Flack parameter: −0.007 (9)

### 

Data collection: *CrysAlis CCD* (Oxford Diffraction, 2006 ▶); cell refinement: *CrysAlis RED* (Oxford Diffraction, 2006 ▶); data reduction: *CrysAlis RED*; program(s) used to solve structure: *SHELXS97* (Sheldrick, 2008 ▶); program(s) used to refine structure: *SHELXL97* (Sheldrick, 2008 ▶); molecular graphics: *ORTEPII* (Johnson, 1976 ▶); software used to prepare material for publication: *SHELXL97*.

## Supplementary Material

Crystal structure: contains datablocks I, global. DOI: 10.1107/S1600536808014396/xu2414sup1.cif

Structure factors: contains datablocks I. DOI: 10.1107/S1600536808014396/xu2414Isup2.hkl

Additional supplementary materials:  crystallographic information; 3D view; checkCIF report

## Figures and Tables

**Table 1:** Selected bond lengths (Å)

V27*A*—O7*A*	1.908 (4)
V27*A*—O28*A*	1.607 (4)
V27*A*—O29*A*	1.703 (4)
V27*A*—O29	2.263 (4)
V27*A*—N11*A*	2.163 (5)
V27*A*—N14*A*	2.147 (5)
V27—O7	1.904 (4)
V27—O28	1.610 (4)
V27—O29	1.694 (3)
V27—O29*A*	2.247 (4)
V27—N11	2.179 (5)
V27—N14	2.135 (5)

**Table 2:** Hydrogen-bond geometry (Å, °)

*D*—H⋯*A*	*D*—H	H⋯*A*	*D*⋯*A*	*D*—H⋯*A*
N14—H14*A*⋯O7*A*	0.92	2.20	2.946 (6)	138
N14*A*—H14*D*⋯O7	0.92	2.02	2.855 (6)	151
O1*W*—H1*W*1⋯O29*A*	0.84	2.14	2.795 (6)	134
O2*W*—H1*W*2⋯O29	0.84	2.04	2.834 (7)	158
C3—H3⋯O1*W*^i^	0.95	2.52	3.431 (8)	160
C3*A*—H3*A*⋯O2*W*^ii^	0.95	2.52	3.424 (9)	159
C10*A*—H10*A*⋯O28^iii^	0.95	2.48	3.075 (7)	120
C13*A*—H13*A*⋯O29	1.00	2.53	3.057 (7)	113

Symmetry codes: (i) 
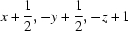
; (ii) 
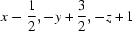
; (iii) 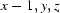.

**Fig. 1.**
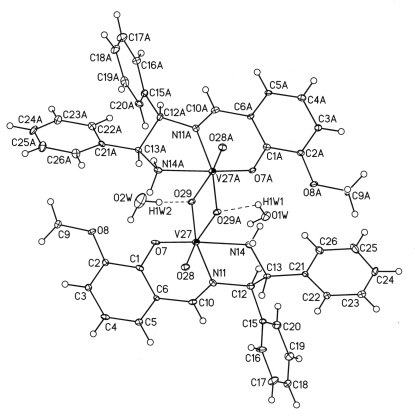
ORTEPII plot (Johnson, 1976) of the title complex, with displacement ellipsoids drawn at the 15% probability level and H atoms are shown as small spheres of arbitrary radius.

**Fig. 2.**
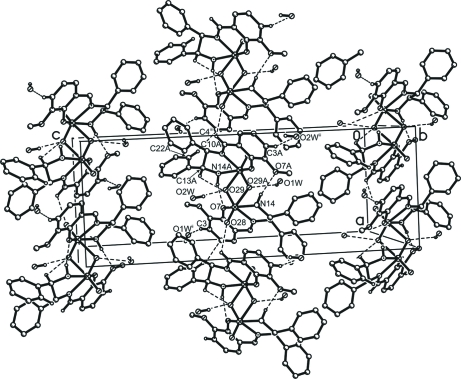
The structure of (I) with water molecules showing hydrogen bonding arrangement.

## supplementary crystallographic information

### Comment

The discovery of vanadium in active sites of biological systems of nitrogenase (Robinson *et al.*, 1986) and bromoperoxidase (Vilter, 1984) and recognition of its environment increased the interest in the vanadium complexes with ligands bearing oxygen and nitrogen atoms for mimicking the biological activity in natural systems. Structural models for the active site in haloperoxidases have already been reported (Gruning & Rehder, 2000; Casny & Rehder, 2001; Kimblin *et al.*, 2002). Recently, it has been established that vanadium(V) complexes with Schiff bases, which are excellent models for active sites of vanadium containing haloperoxidases, are able to catalyze the oxidation of organic sulfides to the corresponding sulfoxides (Kwiatkowski *et al.*, 2003, 2007; Romanowski *et al.*, 2008). Vanadium haloperoxidases catalyze the oxidation of halides in the presence of hydrogen peroxide to highly reactive intermediate, a hypohalous acid, which may react either with suitable nucleophilic acceptor, if present, forming a halogenated compound or with hydrogen peroxide yielding ^1^O_2_ (Wever & Hemrika, 1997). Lately, it has been shown that vanadium bromoperoxidase from marine red algae can catalyze the bromination and cyclization of terpene substrates or the selective sulfoxidation of sulfides to the sulfoxides (Butler & Carter-Franklin, 2004).

The structure of (I) indicates that each vanadium atom is six-coordinated. The vanadium atoms V27 and V27A are joined together through two oxygen bridges, O29 and O29A being the bridging atoms coordinated to vanadium atoms at short and long distances (Table 1 and Fig. 1). The coordination sphere around each vanadium atom is composed of phenolate oxygen atoms (O7 or O7A) and of primary amine nitrogen atom (N14 or N14A) occupying axial positions and of imine nitrogen atom (N11 or N11A), two strongly coordinated terminal oxo (O28, O29 or O28A, O29A) and one weakly associated oxo group (O29A or O29) derived from the neighbouring VO_2_ unit defining the equatorial plane. The polyhedron that describes the quadrilateral core consisting of the V_2_O_2_ unit and the spatial arrangement of remaining coordinated atoms resembles two edge shared octahedrons that are significantly distorted. The V27=O28 and V27A=O28A bond lengths of 1.616 (4) and 1.612 (4) Å respectively compare well with the distances between vanadium and the doubly bonded oxygen atoms (Root *et al.*, 1993; Romanowski *et al.*, 2008). The V27—O29 and V27A—O29A bonds (average 1.696 (4) Å) are longer than V27—O28 and V27A—O28A bonds (average 1.614 (4) Å) due to involvement of O29 and O29A atoms in V27—V27A bridging and hydrogen bonding interactions with water molecules. The V27—V27A distance of 3.057 (2) Å is much shorter than the V(V)—V(V) distance of 3.204 Å found in similar complex (Romanowski *et al.*, 2008). The O28—V27—O29 and O28A—V27A—O29A angles of 105.6 (2)° and 104.6 (2)° respectively indicate significant double bond character of these bonds (Colpas *et al.*, 1994) and are close to other *cis*-VO_2_ units (Li *et al.*, 1988). The two five-membered chelate rings adopt twisted conformations, while in similar dimeric vanadium(V) complex adopt differently puckered envelope conformations (Romanowski *et al.*, 2008). The rings defined by V27, N11, C12, C13, N14 and V27A, N11A, C12A, C13A, N14A choose twisted conformation on C12, C13 and C12A, C13A, respectively.

The crystal structure of (I) is stabilized by intermolecular hydrogen bonds linking dimeric molecules both directly by N—H···O interactions and indirectly through water molecules by O···H—O—H interactions (Fig. 2, Table 2).

### Experimental

The complex (I) were obtained in a template/complexation reactions analogous to those described for preparation of dioxovanadium(V) complexes with Schiff base ligands (Kwiatkowski *et al.*, 2003). A solution of 1 mmol of (*R*,*R*)-(+)-1,2-diphenyl-1,2-diaminoethane in 10 ml of absolute ethanol was added under stirring to a freshly filtered solution of vanadium(V) oxytriethoxide (1 mmol) in 50 ml of absolute EtOH producing a yellow suspension of the intermediate. 3-Methoxysalicylaldehyde (1 mmol) dissolved in absolute EtOH was added to the aforementioned suspension. After refluxing (70 ml) of the resulting mixture for 2 h and its cooling to room temperature the separated solids were filtered off, washed several times with EtOH, recrystallized from DMSO-EtOH mixture and dried over molecular sieves.

### Refinement

All C bonded H atoms were positioned geometrically and refined using a riding model, with C–H distances of 0.93–1.00 Å and with *U*_iso_(H) = 1.2*U*_eq_(C) (C–H = 0.96 Å and *U*_iso_(H) = 1.5*U*_eq_(C) for the methyl group). All remaining H atoms were found in difference Fourier maps and their positions were refined initially with O–H and N–H bond lengths restricted to be 0.92 and 0.84 Å, respectively. In the final stages of refinement these H atoms were constrained to ride on their parent atoms using AFIX 23 and AFIX 3, respectively; (*U*_iso_(H) = 1.2*U*_eq_(N) and *U*_iso_(H) = 1.5*U*_eq_(O)). One H atom from each water molecule is not involved in any hydrogen bonds; an observation confirmed by its IR spectrum which shows a single sharp band at 935 cm^-1^.

### Crystal data

**Table:**

[V_2_(C_22_H_21_N_2_O_2_)_2_O_4_]·2H_2_O	*F*_000_ = 1856
*M**_r_* = 892.73	*D*_x_ = 1.471 Mg m^−^^3^
Orthorhombic, *P*2_1_2_1_2_1_	Cu *K*α radiation λ = 1.5418 Å
Hall symbol: P 2ac 2ab	Cell parameters from 12243 reflections
*a* = 9.328 (4) Å	θ = 3–70º
*b* = 16.950 (7) Å	µ = 4.44 mm^−^^1^
*c* = 25.490 (10) Å	*T* = 100 (2) K
*V* = 4030 (3) Å^3^	Multifaced, violet
*Z* = 4	0.14 × 0.12 × 0.09 mm

### Data collection

**Table:**

Oxford Xcalibur PX κ-geometry diffractometer with CCD area-detector	7083 independent reflections
Radiation source: fine-focus sealed tube	3677 reflections with *I* > 2σ(*I*)
Monochromator: graphite	*R*_int_ = 0.066
*T* = 100(2) K	θ_max_ = 76.5º
ω and φ scans	θ_min_ = 3.1º
Absorption correction: analytical(CrysAlis RED; Oxford Diffraction, 2006)	*h* = −10→11
*T*_min_ = 0.627, *T*_max_ = 0.768	*k* = −19→20
27320 measured reflections	*l* = −20→31

### Refinement

**Table:**

Refinement on *F*^2^	Hydrogen site location: inferred from neighbouring sites
Least-squares matrix: full	H-atom parameters constrained
*R*[*F*^2^ > 2σ(*F*^2^)] = 0.048	*w* = 1/[σ^2^(*F*_o_^2^) + (0.009*P*)^2^] where *P* = (*F*_o_^2^ + 2*F*_c_^2^)/3
*wR*(*F*^2^) = 0.069	(Δ/σ)_max_ = 0.004
*S* = 1.01	Δρ_max_ = 0.64 e Å^−^^3^
7083 reflections	Δρ_min_ = −0.39 e Å^−^^3^
541 parameters	Extinction correction: none
Primary atom site location: structure-invariant direct methods	Absolute structure: Flack (1983), 2905 Friedel pairs
Secondary atom site location: difference Fourier map	Flack parameter: −0.007 (9)

### Special details

**Table:**

Geometry. All e.s.d.'s (except the e.s.d. in the dihedral angle between two l.s. planes) are estimated using the full covariance matrix. The cell e.s.d.'s are taken into account individually in the estimation of e.s.d.'s in distances, angles and torsion angles; correlations between e.s.d.'s in cell parameters are only used when they are defined by crystal symmetry. An approximate (isotropic) treatment of cell e.s.d.'s is used for estimating e.s.d.'s involving l.s. planes.
Refinement. Refinement of *F*^2^ against ALL reflections. The weighted *R*-factor *wR* and goodness of fit *S* are based on *F*^2^, conventional *R*-factors *R* are based on *F*, with *F* set to zero for negative *F*^2^. The threshold expression of *F*^2^ > σ(*F*^2^) is used only for calculating *R*-factors(gt) *etc*. and is not relevant to the choice of reflections for refinement. *R*-factors based on *F*^2^ are statistically about twice as large as those based on *F*, and *R*- factors based on ALL data will be even larger.

### Fractional atomic coordinates and isotropic or equivalent isotropic displacement parameters (Å^2^)

**Table:**

	*x*	*y*	*z*	*U*_iso_*/*U*_eq_	
V27A	0.32884 (10)	0.48070 (7)	0.48510 (4)	0.0321 (3)	
V27	0.64395 (9)	0.51515 (7)	0.50926 (4)	0.0315 (3)	
O28A	0.2087 (4)	0.4322 (2)	0.45349 (16)	0.0364 (12)	
O28	0.7674 (4)	0.5616 (2)	0.54082 (16)	0.0351 (12)	
O29A	0.4860 (4)	0.4571 (3)	0.45501 (15)	0.0319 (12)	
O29	0.4883 (3)	0.5398 (3)	0.53930 (15)	0.0292 (11)	
O7A	0.3123 (4)	0.5855 (2)	0.45846 (15)	0.0300 (11)	
C1A	0.2145 (6)	0.6399 (4)	0.4629 (2)	0.0276 (16)	
C2A	0.2227 (6)	0.7066 (4)	0.4286 (2)	0.0267 (16)	
C3A	0.1167 (6)	0.7649 (4)	0.4295 (2)	0.0291 (17)	
H3A	0.1202	0.8072	0.4051	0.035*	
C4A	0.0064 (6)	0.7611 (4)	0.4659 (3)	0.0320 (18)	
H4A	−0.0659	0.8006	0.4664	0.038*	
C5A	0.0025 (5)	0.7001 (4)	0.5010 (2)	0.0263 (17)	
H5A	−0.0716	0.6987	0.5266	0.032*	
C6A	0.1035 (5)	0.6405 (3)	0.5004 (2)	0.0211 (14)	
O8A	0.3392 (4)	0.7063 (3)	0.39578 (15)	0.0332 (12)	
C9A	0.3526 (7)	0.7745 (4)	0.3617 (2)	0.0395 (19)	
H9D	0.4398	0.7695	0.3405	0.059*	
H9E	0.3584	0.8225	0.3831	0.059*	
H9F	0.2689	0.7776	0.3386	0.059*	
C10A	0.0956 (6)	0.5785 (4)	0.5396 (2)	0.0296 (17)	
H10A	0.0219	0.5835	0.5651	0.036*	
N11A	0.1760 (5)	0.5186 (3)	0.54371 (16)	0.0256 (12)	
C12A	0.1482 (5)	0.4612 (3)	0.58662 (19)	0.0311 (14)	
H12A	0.0870	0.4183	0.5715	0.037*	
C15A	0.0728 (6)	0.4914 (4)	0.6338 (2)	0.0291 (16)	
C20A	0.1342 (6)	0.5481 (4)	0.6645 (2)	0.0367 (17)	
H20A	0.2260	0.5681	0.6554	0.044*	
C19A	0.0649 (7)	0.5771 (4)	0.7088 (2)	0.052 (2)	
H19A	0.1090	0.6169	0.7295	0.063*	
C18A	−0.0669 (8)	0.5481 (4)	0.7227 (3)	0.053 (2)	
H18A	−0.1116	0.5662	0.7539	0.063*	
C17A	−0.1363 (7)	0.4922 (5)	0.6913 (2)	0.057 (2)	
H17A	−0.2293	0.4731	0.6996	0.068*	
C16A	−0.0610 (6)	0.4657 (4)	0.6467 (2)	0.043 (2)	
H16A	−0.1058	0.4280	0.6245	0.051*	
C13A	0.2980 (6)	0.4236 (3)	0.5991 (2)	0.0299 (16)	
H13A	0.3623	0.4664	0.6124	0.036*	
N14A	0.3554 (5)	0.3965 (3)	0.54722 (18)	0.0324 (14)	
H14C	0.3097	0.3504	0.5380	0.039*	
H14D	0.4514	0.3854	0.5508	0.039*	
C21A	0.2923 (6)	0.3601 (4)	0.6398 (2)	0.0282 (17)	
C26A	0.3591 (7)	0.3697 (4)	0.6877 (2)	0.0386 (16)	
H26A	0.4120	0.4167	0.6937	0.046*	
C25A	0.3528 (8)	0.3160 (4)	0.7261 (2)	0.050 (2)	
H25A	0.4012	0.3243	0.7585	0.060*	
C24A	0.2750 (7)	0.2486 (4)	0.7177 (3)	0.051 (2)	
H24A	0.2692	0.2105	0.7449	0.061*	
C23A	0.2058 (6)	0.2345 (4)	0.6716 (2)	0.0465 (18)	
H23A	0.1550	0.1867	0.6659	0.056*	
C22A	0.2121 (6)	0.2926 (4)	0.6331 (2)	0.0440 (19)	
H22A	0.1599	0.2856	0.6015	0.053*	
O7	0.6590 (4)	0.4115 (2)	0.53751 (16)	0.0315 (11)	
C1	0.7654 (6)	0.3571 (4)	0.5352 (2)	0.0274 (16)	
C2	0.7597 (6)	0.2964 (4)	0.5720 (2)	0.0259 (16)	
C3	0.8644 (6)	0.2387 (4)	0.5706 (2)	0.0292 (16)	
H3	0.8626	0.1971	0.5955	0.035*	
C4	0.9722 (6)	0.2413 (4)	0.5331 (3)	0.038 (2)	
H4	1.0444	0.2017	0.5328	0.046*	
C5	0.9748 (6)	0.3004 (4)	0.4967 (2)	0.0367 (18)	
H5	1.0485	0.3014	0.4709	0.044*	
C6	0.8726 (6)	0.3580 (3)	0.4969 (2)	0.0224 (14)	
O8	0.6476 (4)	0.2980 (3)	0.60580 (15)	0.0332 (12)	
C9	0.6331 (6)	0.2312 (4)	0.6400 (2)	0.043 (2)	
H9A	0.5482	0.2380	0.6623	0.065*	
H9B	0.7186	0.2267	0.6622	0.065*	
H9C	0.6225	0.1831	0.6190	0.065*	
C10	0.8681 (6)	0.4112 (4)	0.4535 (2)	0.0279 (16)	
H10	0.9346	0.4019	0.4260	0.033*	
N11	0.7841 (4)	0.4701 (3)	0.44777 (18)	0.0287 (13)	
C12	0.7728 (5)	0.5126 (3)	0.39691 (19)	0.0315 (14)	
H12	0.6891	0.4897	0.3777	0.038*	
C15	0.9029 (5)	0.5043 (4)	0.3612 (2)	0.0243 (16)	
C20	0.8946 (7)	0.4590 (4)	0.3158 (2)	0.044 (2)	
H20	0.8083	0.4318	0.3078	0.053*	
C19	1.0104 (7)	0.4531 (4)	0.2821 (3)	0.049 (2)	
H19	1.0040	0.4221	0.2511	0.059*	
C18	1.1345 (7)	0.4925 (4)	0.2941 (2)	0.0430 (18)	
H18	1.2135	0.4895	0.2707	0.052*	
C17	1.1468 (6)	0.5357 (4)	0.3387 (2)	0.0507 (19)	
H17	1.2337	0.5622	0.3471	0.061*	
C16	1.0299 (6)	0.5401 (4)	0.3714 (2)	0.046 (2)	
H16	1.0386	0.5699	0.4028	0.055*	
C13	0.7381 (5)	0.5996 (3)	0.4075 (2)	0.0260 (15)	
H13	0.8267	0.6249	0.4218	0.031*	
N14	0.6256 (5)	0.6028 (3)	0.44947 (18)	0.0320 (14)	
H14A	0.5371	0.5978	0.4339	0.038*	
H14B	0.6290	0.6517	0.4651	0.038*	
C21	0.7018 (6)	0.6405 (4)	0.3576 (2)	0.0288 (17)	
C26	0.5853 (6)	0.6156 (4)	0.3278 (2)	0.0420 (19)	
H26	0.5277	0.5736	0.3405	0.050*	
C25	0.5504 (7)	0.6501 (4)	0.2800 (2)	0.0512 (19)	
H25	0.4693	0.6337	0.2602	0.061*	
C24	0.6411 (8)	0.7102 (5)	0.2627 (2)	0.056 (2)	
H24	0.6225	0.7330	0.2293	0.067*	
C23	0.7545 (7)	0.7383 (5)	0.2906 (3)	0.068 (3)	
H23	0.8116	0.7805	0.2778	0.082*	
C22	0.7839 (6)	0.7026 (4)	0.3390 (2)	0.050 (2)	
H22	0.8617	0.7214	0.3595	0.060*	
O1W	0.4465 (5)	0.4178 (3)	0.3495 (2)	0.0770 (18)	
H1W1	0.4121	0.4397	0.3762	0.115*	
H2W1	0.4821	0.3734	0.3564	0.115*	
O2W	0.5401 (7)	0.5739 (4)	0.6465 (2)	0.132 (3)	
H1W2	0.5292	0.5769	0.6138	0.199*	
H2W2	0.6260	0.5672	0.6553	0.199*	

### Atomic displacement parameters (Å^2^)

**Table:**

	*U*^11^	*U*^22^	*U*^33^	*U*^12^	*U*^13^	*U*^23^
V27A	0.0266 (6)	0.0352 (7)	0.0346 (6)	0.0044 (5)	0.0053 (5)	0.0018 (7)
V27	0.0244 (5)	0.0340 (7)	0.0361 (6)	0.0034 (5)	0.0068 (5)	0.0017 (6)
O28A	0.030 (2)	0.039 (3)	0.040 (3)	−0.003 (2)	−0.002 (2)	−0.001 (2)
O28	0.027 (2)	0.042 (3)	0.036 (3)	−0.008 (2)	0.002 (2)	−0.010 (2)
O29A	0.029 (2)	0.029 (3)	0.038 (3)	0.003 (2)	0.011 (2)	−0.003 (2)
O29	0.024 (2)	0.028 (3)	0.036 (3)	0.004 (2)	0.0124 (19)	−0.001 (2)
O7A	0.022 (2)	0.034 (3)	0.035 (3)	0.002 (2)	0.012 (2)	0.005 (2)
C1A	0.028 (4)	0.025 (4)	0.030 (4)	0.000 (3)	−0.002 (3)	−0.006 (3)
C2A	0.020 (3)	0.028 (4)	0.032 (4)	−0.005 (3)	−0.001 (3)	−0.001 (4)
C3A	0.025 (3)	0.029 (4)	0.033 (4)	−0.001 (3)	−0.006 (3)	0.004 (3)
C4A	0.025 (3)	0.039 (5)	0.031 (4)	0.004 (3)	0.001 (3)	−0.008 (4)
C5A	0.016 (3)	0.035 (5)	0.028 (4)	0.005 (3)	0.007 (3)	−0.002 (4)
C6A	0.017 (3)	0.024 (4)	0.022 (3)	−0.006 (3)	0.005 (3)	−0.004 (3)
O8A	0.024 (2)	0.036 (3)	0.040 (3)	0.001 (2)	0.013 (2)	0.001 (2)
C9A	0.053 (4)	0.036 (5)	0.030 (4)	−0.002 (4)	0.005 (4)	0.017 (3)
C10A	0.026 (3)	0.040 (5)	0.024 (3)	−0.013 (3)	0.004 (3)	−0.014 (3)
N11A	0.028 (3)	0.020 (3)	0.028 (3)	0.005 (3)	−0.001 (2)	−0.004 (3)
C12A	0.034 (3)	0.034 (4)	0.026 (3)	0.003 (3)	0.006 (3)	0.009 (3)
C15A	0.031 (3)	0.031 (5)	0.025 (3)	0.001 (3)	0.002 (3)	−0.001 (3)
C20A	0.030 (4)	0.044 (4)	0.036 (4)	0.007 (3)	0.002 (3)	−0.007 (3)
C19A	0.068 (5)	0.052 (5)	0.036 (4)	0.026 (4)	−0.001 (4)	−0.004 (4)
C18A	0.061 (5)	0.058 (6)	0.039 (5)	0.025 (4)	0.026 (4)	0.003 (4)
C17A	0.054 (4)	0.072 (6)	0.044 (4)	0.011 (5)	0.017 (4)	−0.002 (5)
C16A	0.035 (4)	0.050 (5)	0.044 (5)	−0.001 (4)	0.004 (3)	0.011 (4)
C13A	0.024 (3)	0.035 (4)	0.030 (4)	0.009 (3)	0.001 (3)	0.006 (3)
N14A	0.025 (3)	0.038 (4)	0.034 (3)	0.006 (3)	0.003 (3)	−0.006 (3)
C21A	0.023 (3)	0.030 (4)	0.032 (4)	0.013 (3)	0.000 (3)	−0.002 (3)
C26A	0.051 (4)	0.024 (4)	0.040 (4)	0.004 (4)	0.000 (4)	−0.001 (3)
C25A	0.073 (6)	0.046 (5)	0.031 (4)	0.001 (5)	0.007 (4)	−0.005 (4)
C24A	0.057 (5)	0.060 (6)	0.037 (5)	0.013 (5)	0.027 (4)	0.014 (4)
C23A	0.045 (4)	0.047 (5)	0.048 (4)	−0.004 (4)	0.004 (3)	0.012 (4)
C22A	0.038 (4)	0.049 (5)	0.045 (4)	−0.002 (3)	−0.004 (3)	0.005 (4)
O7	0.016 (2)	0.035 (3)	0.044 (3)	0.008 (2)	0.005 (2)	−0.006 (2)
C1	0.018 (3)	0.031 (4)	0.033 (4)	−0.002 (3)	−0.006 (3)	−0.008 (4)
C2	0.026 (4)	0.020 (4)	0.032 (4)	−0.001 (3)	−0.004 (3)	0.000 (4)
C3	0.032 (4)	0.028 (4)	0.027 (4)	−0.005 (4)	−0.002 (3)	0.001 (3)
C4	0.022 (3)	0.043 (5)	0.050 (5)	0.013 (4)	0.007 (3)	−0.006 (4)
C5	0.036 (4)	0.041 (5)	0.033 (5)	0.004 (4)	0.008 (4)	0.003 (4)
C6	0.019 (3)	0.020 (3)	0.028 (4)	0.008 (3)	0.000 (3)	0.005 (3)
O8	0.025 (2)	0.040 (3)	0.035 (3)	−0.001 (2)	0.015 (2)	0.003 (2)
C9	0.041 (4)	0.056 (6)	0.033 (4)	0.003 (4)	0.005 (3)	0.011 (4)
C10	0.022 (3)	0.029 (4)	0.033 (4)	0.003 (3)	0.009 (3)	−0.010 (3)
N11	0.024 (3)	0.031 (3)	0.031 (3)	0.002 (3)	−0.005 (2)	−0.005 (3)
C12	0.023 (3)	0.046 (4)	0.025 (3)	−0.006 (3)	0.002 (2)	−0.005 (3)
C15	0.019 (3)	0.030 (4)	0.024 (3)	0.002 (3)	0.003 (2)	−0.003 (3)
C20	0.036 (4)	0.058 (5)	0.038 (4)	0.001 (4)	0.000 (3)	0.000 (4)
C19	0.048 (4)	0.068 (6)	0.031 (4)	0.016 (4)	0.007 (4)	−0.013 (4)
C18	0.039 (4)	0.053 (5)	0.037 (4)	0.009 (4)	0.014 (3)	0.009 (4)
C17	0.045 (4)	0.064 (5)	0.043 (4)	−0.009 (4)	0.019 (4)	−0.010 (4)
C16	0.019 (3)	0.075 (6)	0.044 (4)	0.002 (4)	0.006 (3)	−0.019 (4)
C13	0.021 (3)	0.029 (4)	0.028 (3)	0.001 (3)	0.006 (3)	−0.001 (3)
N14	0.029 (3)	0.025 (4)	0.043 (3)	0.007 (3)	0.004 (3)	−0.004 (3)
C21	0.017 (3)	0.046 (5)	0.023 (4)	0.002 (3)	0.002 (3)	0.007 (3)
C26	0.032 (4)	0.062 (5)	0.032 (4)	0.015 (4)	0.005 (3)	0.004 (4)
C25	0.049 (5)	0.067 (5)	0.037 (4)	0.018 (4)	−0.011 (4)	0.000 (4)
C24	0.058 (5)	0.087 (7)	0.023 (4)	0.023 (5)	0.016 (4)	0.019 (4)
C23	0.038 (5)	0.107 (8)	0.060 (6)	0.003 (5)	0.010 (4)	0.037 (5)
C22	0.031 (4)	0.078 (6)	0.040 (4)	−0.001 (4)	−0.006 (3)	0.022 (4)
O1W	0.094 (4)	0.058 (4)	0.078 (5)	0.009 (3)	0.023 (3)	−0.003 (3)
O2W	0.171 (6)	0.149 (7)	0.076 (5)	−0.062 (5)	0.006 (4)	−0.002 (5)

### Geometric parameters (Å, °)

**Table:**

V27A—O7A	1.908 (4)	C24A—H24A	0.9500
V27A—O28A	1.607 (4)	C23A—C22A	1.391 (7)
V27A—O29A	1.703 (4)	C23A—H23A	0.9500
V27A—O29	2.263 (4)	C22A—H22A	0.9500
V27A—N11A	2.163 (5)	O7—C1	1.356 (7)
V27A—N14A	2.147 (5)	C1—C2	1.393 (8)
V27A—V27	3.0595 (17)	C1—C6	1.398 (7)
V27—O7	1.904 (4)	C2—O8	1.356 (6)
V27—O28	1.610 (4)	C2—C3	1.382 (8)
V27—O29	1.694 (3)	C3—C4	1.388 (8)
V27—O29A	2.247 (4)	C3—H3	0.9500
V27—N11	2.179 (5)	C4—C5	1.367 (8)
V27—N14	2.135 (5)	C4—H4	0.9500
O7A—C1A	1.302 (7)	C5—C6	1.365 (8)
C1A—C6A	1.409 (8)	C5—H5	0.9500
C1A—C2A	1.431 (8)	C6—C10	1.428 (7)
C2A—O8A	1.371 (6)	O8—C9	1.437 (6)
C2A—C3A	1.399 (8)	C9—H9A	0.9800
C3A—C4A	1.386 (8)	C9—H9B	0.9800
C3A—H3A	0.9500	C9—H9C	0.9800
C4A—C5A	1.369 (8)	C10—N11	1.276 (7)
C4A—H4A	0.9500	C10—H10	0.9500
C5A—C6A	1.382 (7)	N11—C12	1.487 (6)
C5A—H5A	0.9500	C12—C15	1.523 (6)
C6A—C10A	1.453 (8)	C12—C13	1.535 (7)
O8A—C9A	1.451 (6)	C12—H12	1.0000
C9A—H9D	0.9800	C15—C16	1.357 (7)
C9A—H9E	0.9800	C15—C20	1.391 (7)
C9A—H9F	0.9800	C20—C19	1.383 (8)
C10A—N11A	1.267 (7)	C20—H20	0.9500
C10A—H10A	0.9500	C19—C18	1.371 (8)
N11A—C12A	1.487 (6)	C19—H19	0.9500
C12A—C15A	1.485 (7)	C18—C17	1.359 (7)
C12A—C13A	1.568 (7)	C18—H18	0.9500
C12A—H12A	1.0000	C17—C16	1.374 (7)
C15A—C16A	1.363 (7)	C17—H17	0.9500
C15A—C20A	1.365 (8)	C16—H16	0.9500
C20A—C19A	1.391 (7)	C13—C21	1.487 (7)
C20A—H20A	0.9500	C13—N14	1.500 (6)
C19A—C18A	1.370 (8)	C13—H13	1.0000
C19A—H19A	0.9500	N14—H14A	0.9200
C18A—C17A	1.399 (9)	N14—H14B	0.9200
C18A—H18A	0.9500	C21—C22	1.386 (8)
C17A—C16A	1.411 (8)	C21—C26	1.391 (7)
C17A—H17A	0.9500	C26—C25	1.389 (8)
C16A—H16A	0.9500	C26—H26	0.9500
C13A—C21A	1.497 (8)	C25—C24	1.396 (9)
C13A—N14A	1.499 (6)	C25—H25	0.9500
C13A—H13A	1.0000	C24—C23	1.361 (9)
N14A—H14C	0.9200	C24—H24	0.9500
N14A—H14D	0.9200	C23—C22	1.401 (9)
C21A—C22A	1.377 (8)	C23—H23	0.9500
C21A—C26A	1.381 (8)	C22—H22	0.9500
C26A—C25A	1.339 (7)	O1W—H1W1	0.8401
C26A—H26A	0.9500	O1W—H2W1	0.8402
C25A—C24A	1.369 (9)	O2W—H1W2	0.8405
C25A—H25A	0.9500	O2W—H2W2	0.8402
C24A—C23A	1.362 (9)		
			
O28A—V27A—O29A	104.7 (2)	C13A—N14A—H14D	108.8
O28A—V27A—O7A	103.99 (19)	V27A—N14A—H14D	108.8
O29A—V27A—O7A	97.37 (19)	H14C—N14A—H14D	107.7
O28A—V27A—N14A	96.3 (2)	C22A—C21A—C26A	116.9 (6)
O29A—V27A—N14A	94.39 (19)	C22A—C21A—C13A	122.1 (6)
O7A—V27A—N14A	153.03 (17)	C26A—C21A—C13A	120.8 (6)
O28A—V27A—N11A	92.24 (19)	C25A—C26A—C21A	123.2 (7)
O29A—V27A—N11A	161.58 (18)	C25A—C26A—H26A	118.4
O7A—V27A—N11A	85.16 (17)	C21A—C26A—H26A	118.4
N14A—V27A—N11A	76.36 (18)	C26A—C25A—C24A	118.4 (7)
O28A—V27A—O29	172.23 (19)	C26A—C25A—H25A	120.8
O29A—V27A—O29	79.23 (15)	C24A—C25A—H25A	120.8
O7A—V27A—O29	81.88 (16)	C23A—C24A—C25A	122.2 (7)
N14A—V27A—O29	76.60 (17)	C23A—C24A—H24A	118.9
N11A—V27A—O29	83.10 (14)	C25A—C24A—H24A	118.9
O28A—V27A—V27	150.30 (15)	C24A—C23A—C22A	117.6 (7)
O29A—V27A—V27	46.21 (14)	C24A—C23A—H23A	121.2
O7A—V27A—V27	88.38 (12)	C22A—C23A—H23A	121.2
N14A—V27A—V27	82.40 (13)	C21A—C22A—C23A	121.6 (6)
N11A—V27A—V27	115.95 (12)	C21A—C22A—H22A	119.2
O29—V27A—V27	33.01 (9)	C23A—C22A—H22A	119.2
O28—V27—O29	105.49 (19)	C1—O7—V27	131.7 (4)
O28—V27—O7	102.1 (2)	O7—C1—C2	116.4 (6)
O29—V27—O7	96.86 (19)	O7—C1—C6	123.1 (6)
O28—V27—N14	94.2 (2)	C2—C1—C6	120.4 (6)
O29—V27—N14	94.74 (19)	O8—C2—C3	125.2 (6)
O7—V27—N14	156.62 (18)	O8—C2—C1	116.3 (5)
O28—V27—N11	95.84 (19)	C3—C2—C1	118.5 (6)
O29—V27—N11	157.77 (18)	C2—C3—C4	120.5 (6)
O7—V27—N11	84.50 (18)	C2—C3—H3	119.7
N14—V27—N11	77.22 (19)	C4—C3—H3	119.7
O28—V27—O29A	171.98 (18)	C5—C4—C3	120.2 (6)
O29—V27—O29A	79.87 (15)	C5—C4—H4	119.9
O7—V27—O29A	82.93 (17)	C3—C4—H4	119.9
N14—V27—O29A	79.22 (17)	C6—C5—C4	120.6 (6)
N11—V27—O29A	78.29 (14)	C6—C5—H5	119.7
O28—V27—V27A	151.77 (14)	C4—C5—H5	119.7
O29—V27—V27A	46.71 (14)	C5—C6—C1	119.7 (6)
O7—V27—V27A	88.33 (12)	C5—C6—C10	118.0 (6)
N14—V27—V27A	84.95 (13)	C1—C6—C10	121.8 (5)
N11—V27—V27A	111.38 (12)	C2—O8—C9	116.3 (5)
O29A—V27—V27A	33.16 (9)	O8—C9—H9A	109.5
V27A—O29A—V27	100.63 (19)	O8—C9—H9B	109.5
V27—O29—V27A	100.27 (19)	H9A—C9—H9B	109.5
C1A—O7A—V27A	133.3 (4)	O8—C9—H9C	109.5
O7A—C1A—C6A	125.3 (6)	H9A—C9—H9C	109.5
O7A—C1A—C2A	117.9 (6)	H9B—C9—H9C	109.5
C6A—C1A—C2A	116.7 (6)	N11—C10—C6	126.9 (5)
O8A—C2A—C3A	125.0 (6)	N11—C10—H10	116.5
O8A—C2A—C1A	114.4 (5)	C6—C10—H10	116.5
C3A—C2A—C1A	120.7 (6)	C10—N11—C12	121.4 (5)
C4A—C3A—C2A	120.2 (6)	C10—N11—V27	124.0 (4)
C4A—C3A—H3A	119.9	C12—N11—V27	114.5 (3)
C2A—C3A—H3A	119.9	N11—C12—C15	114.8 (5)
C5A—C4A—C3A	119.5 (6)	N11—C12—C13	109.1 (4)
C5A—C4A—H4A	120.2	C15—C12—C13	111.2 (5)
C3A—C4A—H4A	120.2	N11—C12—H12	107.1
C4A—C5A—C6A	121.7 (6)	C15—C12—H12	107.1
C4A—C5A—H5A	119.1	C13—C12—H12	107.1
C6A—C5A—H5A	119.1	C16—C15—C20	117.0 (5)
C5A—C6A—C1A	121.0 (6)	C16—C15—C12	122.7 (5)
C5A—C6A—C10A	119.0 (5)	C20—C15—C12	120.3 (5)
C1A—C6A—C10A	120.0 (5)	C19—C20—C15	120.9 (6)
C2A—O8A—C9A	115.5 (5)	C19—C20—H20	119.5
O8A—C9A—H9D	109.5	C15—C20—H20	119.5
O8A—C9A—H9E	109.5	C18—C19—C20	119.0 (6)
H9D—C9A—H9E	109.5	C18—C19—H19	120.5
O8A—C9A—H9F	109.5	C20—C19—H19	120.5
H9D—C9A—H9F	109.5	C17—C18—C19	121.4 (6)
H9E—C9A—H9F	109.5	C17—C18—H18	119.3
N11A—C10A—C6A	127.3 (6)	C19—C18—H18	119.3
N11A—C10A—H10A	116.3	C18—C17—C16	118.0 (6)
C6A—C10A—H10A	116.3	C18—C17—H17	121.0
C10A—N11A—C12A	118.8 (5)	C16—C17—H17	121.0
C10A—N11A—V27A	124.9 (4)	C15—C16—C17	123.6 (6)
C12A—N11A—V27A	115.3 (3)	C15—C16—H16	118.2
C15A—C12A—N11A	116.9 (5)	C17—C16—H16	118.2
C15A—C12A—C13A	113.4 (4)	C21—C13—N14	115.7 (5)
N11A—C12A—C13A	105.0 (4)	C21—C13—C12	110.2 (5)
C15A—C12A—H12A	107.0	N14—C13—C12	107.9 (4)
N11A—C12A—H12A	107.0	C21—C13—H13	107.6
C13A—C12A—H12A	107.0	N14—C13—H13	107.6
C16A—C15A—C20A	118.1 (6)	C12—C13—H13	107.6
C16A—C15A—C12A	121.2 (6)	C13—N14—V27	115.4 (3)
C20A—C15A—C12A	120.6 (5)	C13—N14—H14A	108.4
C15A—C20A—C19A	121.3 (6)	V27—N14—H14A	108.4
C15A—C20A—H20A	119.4	C13—N14—H14B	108.4
C19A—C20A—H20A	119.4	V27—N14—H14B	108.4
C18A—C19A—C20A	120.0 (7)	H14A—N14—H14B	107.5
C18A—C19A—H19A	120.0	C22—C21—C26	118.3 (6)
C20A—C19A—H19A	120.0	C22—C21—C13	121.4 (6)
C19A—C18A—C17A	120.7 (7)	C26—C21—C13	120.3 (6)
C19A—C18A—H18A	119.6	C25—C26—C21	122.4 (6)
C17A—C18A—H18A	119.6	C25—C26—H26	118.8
C18A—C17A—C16A	116.5 (7)	C21—C26—H26	118.8
C18A—C17A—H17A	121.7	C26—C25—C24	116.2 (6)
C16A—C17A—H17A	121.7	C26—C25—H25	121.9
C15A—C16A—C17A	123.3 (7)	C24—C25—H25	121.9
C15A—C16A—H16A	118.4	C23—C24—C25	124.2 (7)
C17A—C16A—H16A	118.4	C23—C24—H24	117.9
C21A—C13A—N14A	113.8 (5)	C25—C24—H24	117.9
C21A—C13A—C12A	113.6 (4)	C24—C23—C22	117.5 (7)
N14A—C13A—C12A	105.3 (4)	C24—C23—H23	121.3
C21A—C13A—H13A	108.0	C22—C23—H23	121.3
N14A—C13A—H13A	108.0	C21—C22—C23	121.4 (6)
C12A—C13A—H13A	108.0	C21—C22—H22	119.3
C13A—N14A—V27A	113.9 (3)	C23—C22—H22	119.3
C13A—N14A—H14C	108.8	H1W1—O1W—H2W1	112.2
V27A—N14A—H14C	108.8	H1W2—O2W—H2W2	112.9
			
O7A—C1A—C2A—O8A	−2.6 (8)	C13A—C21A—C22A—C23A	−179.0 (5)
C6A—C1A—C2A—O8A	174.9 (5)	C24A—C23A—C22A—C21A	3.8 (9)
O7A—C1A—C2A—C3A	176.6 (5)	O7—C1—C2—O8	0.5 (8)
C6A—C1A—C2A—C3A	−5.9 (9)	C6—C1—C2—O8	−176.5 (5)
O8A—C2A—C3A—C4A	−177.2 (6)	O7—C1—C2—C3	178.9 (5)
C1A—C2A—C3A—C4A	3.7 (9)	C6—C1—C2—C3	1.9 (9)
C2A—C3A—C4A—C5A	0.3 (10)	O8—C2—C3—C4	177.8 (6)
C3A—C4A—C5A—C6A	−2.0 (10)	C1—C2—C3—C4	−0.4 (9)
C4A—C5A—C6A—C1A	−0.5 (9)	C2—C3—C4—C5	−0.8 (10)
C4A—C5A—C6A—C10A	178.3 (6)	C3—C4—C5—C6	0.5 (11)
O7A—C1A—C6A—C5A	−178.4 (6)	C4—C5—C6—C1	0.9 (10)
C2A—C1A—C6A—C5A	4.4 (8)	C4—C5—C6—C10	−170.8 (6)
O7A—C1A—C6A—C10A	2.8 (9)	O7—C1—C6—C5	−179.0 (6)
C2A—C1A—C6A—C10A	−174.5 (5)	C2—C1—C6—C5	−2.2 (9)
C3A—C2A—O8A—C9A	2.8 (8)	O7—C1—C6—C10	−7.5 (9)
C1A—C2A—O8A—C9A	−178.1 (5)	C2—C1—C6—C10	169.3 (6)
C5A—C6A—C10A—N11A	177.6 (5)	C3—C2—O8—C9	−4.6 (8)
C1A—C6A—C10A—N11A	−3.5 (9)	C1—C2—O8—C9	173.6 (5)
C6A—C10A—N11A—C12A	−179.0 (5)	C5—C6—C10—N11	−177.3 (6)
C6A—C10A—N11A—V27A	−10.7 (8)	C1—C6—C10—N11	11.1 (10)
C10A—N11A—C12A—C15A	−24.9 (7)	C6—C10—N11—C12	−169.2 (5)
C10A—N11A—C12A—C13A	−151.6 (5)	C6—C10—N11—V27	9.4 (8)
N11A—C12A—C15A—C16A	114.2 (7)	C10—N11—C12—C15	−23.2 (7)
C13A—C12A—C15A—C16A	−123.4 (6)	C10—N11—C12—C13	−148.8 (5)
N11A—C12A—C15A—C20A	−63.5 (7)	N11—C12—C15—C16	−71.8 (8)
C13A—C12A—C15A—C20A	59.0 (8)	C13—C12—C15—C16	52.7 (8)
C16A—C15A—C20A—C19A	2.2 (9)	N11—C12—C15—C20	108.6 (6)
C12A—C15A—C20A—C19A	180.0 (6)	C13—C12—C15—C20	−126.8 (6)
C15A—C20A—C19A—C18A	0.7 (10)	C16—C15—C20—C19	−1.7 (10)
C20A—C19A—C18A—C17A	−3.1 (11)	C12—C15—C20—C19	177.9 (6)
C19A—C18A—C17A—C16A	2.6 (11)	C15—C20—C19—C18	0.1 (10)
C20A—C15A—C16A—C17A	−2.8 (10)	C20—C19—C18—C17	1.4 (11)
C12A—C15A—C16A—C17A	179.5 (6)	C19—C18—C17—C16	−1.1 (11)
C18A—C17A—C16A—C15A	0.4 (11)	C20—C15—C16—C17	2.0 (11)
C15A—C12A—C13A—C21A	54.4 (7)	C12—C15—C16—C17	−177.6 (6)
N11A—C12A—C13A—C21A	−176.8 (5)	C18—C17—C16—C15	−0.6 (11)
C15A—C12A—C13A—N14A	179.5 (5)	N11—C12—C13—C21	−170.4 (4)
N11A—C12A—C13A—N14A	−51.7 (6)	C15—C12—C13—C21	61.9 (6)
C21A—C13A—N14A—V27A	169.5 (4)	N11—C12—C13—N14	−43.1 (5)
C12A—C13A—N14A—V27A	44.5 (5)	C15—C12—C13—N14	−170.8 (4)
O28A—V27A—N14A—C13A	−109.7 (4)	C21—C13—N14—V27	160.3 (4)
O29A—V27A—N14A—C13A	144.9 (4)	C12—C13—N14—V27	36.4 (5)
O7A—V27A—N14A—C13A	29.1 (6)	N14—C13—C21—C22	119.4 (6)
N11A—V27A—N14A—C13A	−18.9 (4)	C12—C13—C21—C22	−117.9 (6)
O29—V27A—N14A—C13A	67.1 (4)	N14—C13—C21—C26	−62.0 (8)
N14A—C13A—C21A—C22A	−60.2 (7)	C12—C13—C21—C26	60.7 (7)
C12A—C13A—C21A—C22A	60.3 (8)	C22—C21—C26—C25	0.9 (9)
N14A—C13A—C21A—C26A	124.8 (6)	C13—C21—C26—C25	−177.7 (6)
C12A—C13A—C21A—C26A	−114.7 (6)	C21—C26—C25—C24	1.6 (9)
C22A—C21A—C26A—C25A	2.3 (10)	C26—C25—C24—C23	−3.2 (10)
C13A—C21A—C26A—C25A	177.5 (6)	C25—C24—C23—C22	2.1 (12)
C21A—C26A—C25A—C24A	−0.8 (11)	C26—C21—C22—C23	−2.1 (10)
C26A—C25A—C24A—C23A	0.7 (11)	C13—C21—C22—C23	176.5 (6)
C25A—C24A—C23A—C22A	−2.2 (10)	C24—C23—C22—C21	0.7 (11)
C26A—C21A—C22A—C23A	−3.8 (9)		

### Hydrogen-bond geometry (Å, °)

**Table:**

*D*—H···*A*	*D*—H	H···*A*	*D*···*A*	*D*—H···*A*
N14—H14A···O7A	0.92	2.20	2.946 (6)	138
N14A—H14D···O7	0.92	2.02	2.855 (6)	151
O1W—H1W1···O29A	0.84	2.14	2.795 (6)	134
O2W—H1W2···O29	0.84	2.04	2.834 (7)	158
C3—H3···O1W^i^	0.95	2.52	3.431 (8)	160
C3A—H3A···O2W^ii^	0.95	2.52	3.424 (9)	159
C10A—H10A···O28^iii^	0.95	2.48	3.075 (7)	120
C13A—H13A···O29	1.00	2.53	3.057 (7)	113

Symmetry codes: (i) *x*+1/2, −*y*+1/2, −*z*+1; (ii) *x*−1/2, −*y*+3/2, −*z*+1; (iii) *x*−1, *y*, *z*.
